# Poster Session I - A173 EN-BLOC RESECTION RATE USING ENDOSCOPIC SUBMUCOSAL DISSECTION (ESD) IN THE MANAGEMENT OF RECTAL POLYPS: A RETROSPECTIVE COHORT STUDY

**DOI:** 10.1093/jcag/gwaf042.173

**Published:** 2026-02-13

**Authors:** M Scaffidi, A Rostom, R Lee, Y Fujiyoshi

**Affiliations:** Gastroenterology, University of Ottawa Faculty of Medicine, Ottawa, ON, Canada; Gastroenterology, University of Ottawa Faculty of Medicine, Ottawa, ON, Canada; Gastroenterology, University of Ottawa Faculty of Medicine, Ottawa, ON, Canada; Gastroenterology, University of Ottawa Faculty of Medicine, Ottawa, ON, Canada

## Abstract

**Background:**

Large colorectal polyps (≥20 mm) carry risk of covert cancer. While endoscopic mucosal resection (EMR) resects most lesions, recurrence can occur. Endoscopic submucosal dissection (ESD) enables en-bloc, margin-controlled excision with lower recurrence. To date, there has been limited uptake of ESD in Canada.

**Aims:**

To describe rectal-ESD outcomes at a Canadian tertiary centre.

**Methods:**

We retrospectively reviewed consecutive rectal ESDs at The Ottawa Hospital (Jan 2019–Apr 2025). Extracted data included age, sex, lesion size, Paris morphology (or multiple morphologies), and ESD technique (conventional, hybrid, pocket/tunnel). The primary outcome was en-bloc resection rate. Secondary outcomes were adverse events (perforation, delayed bleeding, post-ESD syndrome) and 30-day mortality. Rates are n/N (%) and interquartile range (IQR).

**Results:**

Fourteen patients (median age 71.2 years, IQR 65.3–74.5; 50 % female) underwent rectal ESD. Lesions measured 5.0 cm (2.8–7.0) and exhibited ≥ 1 Paris subtype: 0-Is 6 (42.9 %), 0-IIa 6 (42.9 %), 0-Isp 2 (14.3 %). Techniques employed were conventional ESD in 5 cases (35.7 %), hybrid ESD/EMR in 5 cases (35.7 %) and pocket/tunnel ESD in 4 cases (28.6 %). En-bloc resection was achieved in 9/12 evaluable cases (75.0 %; 95 % CI 43.7–92.2). Three perforations occurred (21.4 %; 95 % CI 4.7–50.8), all closed endoscopically with clips/over the scope clip (OTSC), and no 30-day mortality.

**Conclusions:**

Among a cohort of rectal ESD cases, a 75 % en-bloc rate was achieved, with perforations in one-fifth of patients but no surgery or mortality required. Future directions involve prospective series and benchmarking against EMR are needed to inform case selection, technique, and quality targets in Canadian practice.

**Funding Agencies:**

None

